# Fats and oils – a scoping review for Nordic Nutrition Recommendations 2023

**DOI:** 10.29219/fnr.v68.10487

**Published:** 2024-02-09

**Authors:** Fredrik Rosqvist, Sari Niinistö

**Affiliations:** 1 Department of Public Health and Caring Sciences, Clinical Nutrition and Metabolism, Uppsala University, Uppsala, Sweden; 2 Finnish Institute for Health and Welfare, Helsinki, Finland

**Keywords:** fats, oils, butter, olive oil, rapeseed oil, palm oil, vegetable oil, coconut oil, LDL cholesterol, diabetes, cardiovascular disease, mortality, cancer

## Abstract

This scoping review for the Nordic Nutrition Recommendations 2023 summarizes the available evidence on fats and oils from a food level perspective. A literature search for systematic reviews (SRs) and meta-analyses was conducted in PubMed. There are few SRs and meta-analyses available that investigate the association between fats and oils (food level) and health outcomes; the majority report associations at the nutrient level (fatty acid classes). All identified SRs and meta-analyses were of low methodological quality, thus the findings and conclusions presented within this scoping review should be interpreted cautiously. Based on this limited evidence, the following results were indicated: the intake of olive oil may be associated with reduced risk of cardiovascular disease (CVD), type 2 diabetes (T2D), and total mortality in prospective cohort studies. The intake of butter was not associated with the risk of CVD but may be related to slightly lower risk of T2D and higher risk of total mortality in prospective cohort studies. For cancer, the evidence is sparse and primarily based on case-control studies. The intake of olive oil may be associated with reduced risk of cancer, whereas the intake of butter may be associated with increased risk of certain cancer types. Butter increases LDL-cholesterol when compared to virtually all other fats and oils. Palm oil may increase LDL-cholesterol when compared to oils rich in MUFA or PUFA but may not have any effect on glucose or insulin. Coconut oil may increase LDL-cholesterol when compared to other plant oils but may decrease LDL-cholesterol when compared to animal fats rich in SFA. Canola/rapeseed oil may decrease LDL-cholesterol compared to olive oil, sunflower oil and sources of SFA and may also reduce body weight compared to other oils. Olive oil may decrease some inflammation markers but may not have a differential effect on LDL-cholesterol compared to other fats and oils. The effect on risk markers likely differs depending on the type/version of oil, for example, due to the presence of polyphenols, phytosterols and other minor components. Taken together, based on the available evidence, oils rich in unsaturated fat (e.g. olive oil, canola oil) are to be preferred over oils and fats rich in saturated fat (e.g. butter, tropical oils).

## Popular scientific summary

- Fats and oils vary mainly in fatty acid composition but also in their content of polyphenols and other bioactive components.
- Among the Nordic and Baltic countries, the use of fats and oils appears to be highest in Denmark and Finland.
- The intake of olive oil is associated with reduced risk of cardiovascular disease, type 2 diabetes, some types of cancer, and total mortality.
- Butter, palm oil, and coconut oil increase LDL cholesterol levels when compared to plant oils high in unsaturated fatty acids.
- Choosing vegetable oils rich in unsaturated fatty acids, such as olive and rapeseed oil, over tropical plant oils and animal fats high in saturated fatty acids seems most beneficial for health.

**Fig. 1:**
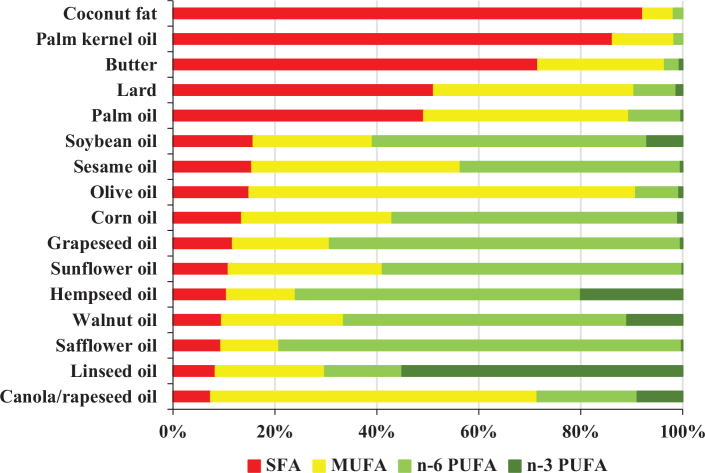
Compositional differences between various fats and oils. Values are taken from the Swedish, Finnish and US food databases which showed the amount of different types of fatty acids as grams per 100 g fat or oil which equals to percentages.

Dietary sources of fat are usually, and conveniently, defined by their fatty acid compositions as saturated (SFA), monounsaturated (MUFA) or polyunsaturated (PUFA) fat rich sources (illustrated in Fig. 1) as the degree of saturation is known to have a meaningful impact on important risk markers (e.g. blood lipids). Although the degree of saturation is likely to be the primary mediator in terms of health effects of dietary fats and oils, it is not the only factor. Vegetable oils may also contain different levels of other bioactive components, such as polyphenols, antioxidants, vitamins and phytosterols, potentially mediating parts of the overall health effect. These bioactive compounds may be more or less preserved depending on the degree of processing (1). Olive oil is a well-known example, where the level of polyphenols may vary widely due to agronomic factors (e.g. ripeness of the olives and cultivation conditions), extraction technology, and mixing of different fractions (2). In general, extra-virgin olive oil has the highest content of polyphenols whereas levels may be very low in refined olive oils. Similarly, the natural content of phytosterols (known to have beneficial effects on blood cholesterol levels) varies markedly between different fats and oils, being markedly higher in, for example, rapeseed and corn oil than in, for example, sunflower, soybean and olive oil (1). However, and similar to polyphenols on olive oil, the levels of phytosterols in oils will be affected by the cultivation conditions, extraction method, and degree of refinement (1). Thus, the overall health effect of various sources of fat may differ, even if the fatty acid composition is similar, due to complex interactions between nutrients, non-nutrients and physical structure (known as food matrix-effects). Furthermore, for some fats and oils, multiple fractions may be obtained. An illustrative example of this is palm oil, consisting of roughly equal parts of saturated and unsaturated fatty acids and is viewed as a source of saturated fat in the diet. However, multiple different fractions can be obtained from palm oil. These fractions have different physicochemical properties and differ markedly in their fatty acid composition (3). For example, compared to ‘palm oil’, ‘palm olein’ has a lower content of palmitic acid (and total SFA) and is semi-solid in room temperature, whereas both ‘super olein’ and ‘top olein’ are liquid in room temperature due to even lower contents of palmitic acid (and total SFA) (3). In contrast to ‘palm oil’, ‘palm stearin’ has a markedly higher content of palmitic acid (and total SFA) and is solid at room temperature (3). In extreme contrast to ‘palm oil’, ‘palm olein’ and ‘palm stearin’, palm kernel fractions (e.g. palm kernel oil and -olein) have a very low content of palmitic acid and is dominated primarily by lauric acid (~45%) but also myristic acid (~15%) (3). Finally, red palm oil (crude/unprocessed) has a high content of carotenoids, antioxidants, vitamin E, and phytosterols but has limited utility and is seldom used. Thus, in order to draw conclusions about the health effects of palm oil compared to other oils, it is important to know which fraction(s) that have been used. Palm oil is a so-called ‘tropical oil’, which means that it is a vegetable oil rich in saturated fat derived from the tropical zone. These vegetable oils (also including e.g. coconut oil) are distinctly different from the so-called ‘non-tropical oils’, which are instead characterized by their low content of saturated fat.

The aim of this scoping review is to describe the totality of evidence for the role of fats and oils for health-related outcomes as a basis for setting and updating food-based dietary guidelines in the Nordic Nutrition Recommendations (NNR) 2023 (Box 1). This scoping review primarily considers results at a food level perspective (i.e. studies reporting on specific food sources of fat, e.g. olive oil). Results from studies reporting at a nutrient level perspective (e.g. saturated fat) are considered in an accompanying paper (4).

*Box 1.* Background papers for Nordic Nutrition Recommendations 2023

- This paper is one of many scoping reviews commissioned as part of the Nordic Nutrition Recommendations 2023 (NNR2023) project (5)
- The papers are included in the extended NNR2023 report but, for transparency, these scoping reviews are also published in Food & Nutrition Research
- The scoping reviews have been peer reviewed by independent experts in the research field according to the standard procedures of the journal
- The scoping reviews have also been subjected to public consultations (see report to be published by the NNR2023 project)
- The NNR2023 committee has served as the editorial board
- While these papers are a main fundament, the NNR2023 committee has the sole responsibility for setting dietary reference values in the NNR2023 project

## Methods

This review follows the protocol developed within the NNR2023 project (5). The sources of evidence used in this review follow the eligibility criteria described previously (6). No *de novo* NNR2023 systematic review (SR) was performed for this paper, and no previously published qualified SRs reporting at a food level perspective were available (5, 7). However, one qualified SR primarily reporting at a nutrient level could be used as supporting evidence for the association between butter and cardiovascular disease (CVD) (8). A literature search for SR and meta-analyses was conducted in PubMed on 16 August 2021 (updated on 31 January 2022) using the following search string (“dietary fat”[Title/Abstract] odds ratio [OR] “dietary fats”[Title/Abstract] OR “vegetable oil”[Title/Abstract] OR “butter”[Title/Abstract] OR “ghee”[Title/Abstract] OR “corn oil”[Title/Abstract] OR “cottonseed oil”[Title/Abstract] OR “canola”[Title/Abstract] OR “olive oil”[Title/Abstract] OR “rapeseed oil”[Title/Abstract] OR “safflower oil”[Title/Abstract] OR “sunflower oil”[Title/Abstract] OR “sesame oil”[Title/Abstract] OR “soybean oil”[Title/Abstract] OR “plant oil”[Title/Abstract] OR “seed oil”[Title/Abstract] OR “cooking oil”[Title/Abstract] OR “margarine”[Title/Abstract] OR “flaxseed oil”[Title/Abstract] OR “palm oil”[Title/Abstract] OR “coconut oil”[Title/Abstract] OR “camelina oil”[Title/Abstract] OR “lard”[Title/Abstract] OR “mayonnaise”[Title/Abstract] OR “dietary fats”[Mesh] OR “plant oils”[Mesh] AND ((meta-analysis[Filter] OR systematicreview[Filter]) AND (humans[Filter]) AND (2011:2021[pdat])). For publications reporting results at both food- and nutrient level, primarily the food level perspective is considered in this paper. The search resulted in 961 hits. Study selection was done in duplicate and consensus was reached through discussion between the authors. The search was repeated without filtering for ‘Humans’ which resulted in an additional three papers of relevance. The quality of the included studies (Table 1) was evaluated using the tool AMSTAR 2, modified for NNR (5). The certainty of evidence was not assessed. Excluded studies are presented in Table 2. The topics discussed in this scoping review are, to a large degree, defined by the topics covered by the SRs. In case of multiple and overlapping SRs, we chose the most recent and most updated SRs over the older, or used both if they were complementary.

**Table 1:** List of included studies

1st author (year)	Exposure	Comparison	Number and design of included studies/comparisons	Main findings/Conclusions	AMSTAR2-NNR rating
***Cardiovascular disease***					
Schwingshackl (2014)	Olive oil	Highest vs. lowest consumption	*n* = 7 cohorts for CV events, *n* = 5 cohorts for CV mortality, *n* = 2 cohorts for stroke, *n* = 4 cohorts for CHD	CV events (RR 0.72 [0.57–0.91], *I*^2^ = 75%). CV mortality (RR 0.70 [0.48–1.03], *I*^2^ = 71%). Stroke (RR 0.60 [0.47–0.77], *I*^2^ = 0%). CHD (RR 0.80 [0.57–1.14], *I*^2^ = 77%)	Critically low confidence
Martinez-Gonzales (2014)	Olive oil	Per 25 g/day increment	*n* = 5 cohorts for CHD, *n* = 3 cohorts for stroke	CHD (RR 0.94 [0.78–1.14], *I*^2^ = 66%). Stroke (RR 0.76 [0.67–0.86], *I*^2^ = 0%)	Low confidence
Pimpin (2016)	Butter	per 14 g/day increment	*n* = 4 cohorts for any CVD, *n* = 3 cohorts for CHD, *n* = 3 cohorts for stroke, *n* = 2 cohorts for total CVD	Any CVD (RR 1.00 [0.98–1.02], *I*^2^ = 0%). CHD (RR 0.99 [0.96–1.03], *I*^2^ = 0%). Stroke (RR 1.01 [0.98–1.03], *I*^2^ = 0%). Total CVD (RR 0.99 [0.96–1.02], *I*^2^ = 0%)	Low confidence
de Goede (2016)	Butter	per 10 g/day	*n* = 4 cohorts for stroke	RR 1.0 (0.99–1.01), *I*^2^ =0%	Critically low confidence
Ismail (2018)	Palm oil	Other oils, soybean oil (5% TFA), soybean oil (22% TFA)	*n* = 1 CC study for MI	OR 1.26 (1.02–1.55) vs. ‘other oils’. OR 1.33 (1.09–1.62) vs. ‘soybean oil (5% TFA)’. OR 1.16 (0.86–1.56) vs. ‘soybean oil (22% TFA)’	High confidence
***Type 2 diabetes***					
Schwingshackl (2017)	Olive oil	High vs. low	*n* = 4 cohorts	RR 0.84 (0.77, 0.92), *I*^2^ = 22%	High confidence
	Olive oil	per 10 g/day increment	*n* = 4 cohorts	RR 0.91 (0.87–0.95)	
Sayon-Orea (2015)	Olive oil	Low-fat diet in RCT studies	*n* = 2 RCTs (PREDIMED) and *n* = 1 cohort	HR 0.60 (0.43–0.85) (in all centers); no association in the cohort study	Critically low confidence
Pimpin (2016)	Butter	per 14 g/day increment	Four publications including *n* = 11 country-specific cohorts	RR 0.96 (0.93–0.99), *I*^2^ = 47%	Low confidence
***Cancer***					
Markellos (2022)	Olive oil	Highest vs. lowest	*n* = 45 (8 cohorts, 37 CC) for any type of cancer, *n* = 14 (3 cohorts, 11 CC) for breast, 15 (2 cohorts, 13 CC) for gastrointestinal, *n* = 6 CC for upper aerodigestive, *n* = 6 CC for urinary tract	Any type (RR 0.69 [0.62–0.77], *I*^2^ = 75%). Breast (RR 0.67 [0.52– 0.86], *I*^2^ = 83%). Gastrointestinal (RR 0.77 [0.66–0.89], *I*^2^ = 41%). Upper aerodigestive (RR 0.74 [0.60–0.91], *I*^2^ = 33%). Urinary tract (RR 0.46 [0.29–0.72], *I*^2^ = 73%).	Low confidence
Xin (2015)	Vegetable oil	Highest vs. lowest; Per 10 g/day increment	*n* = 16 (5 cohorts and 11 CC); *n* = 12 in dose-response analysis	Breast highest vs. lowest (OR 0.88 [0.77–1.01], *I*^2^ = 74%). Breast per 10 g/day increment (OR 0.98 [0.95–1.01]).	Low confidence
Li (2018)	Butter	Highest vs. lowest	*n* = 2 cohorts	Endometrial RR 1.14 (1.03–1.26), *I*^2^ = 3%	Critically low confidence
Wang (2016)	Butter	Highest vs. lowest	*n* = 4 CC	Non-Hodgkin lymphoma RR 1.31 (1.04–1.65), *I*^2^ = 37%	Critically low confidence
Li (2016)	Butter	High vs. low	*n* = 4 CC	Esophageal squamous cell carcinoma RR 1.77 (0.84–3.75), *I*^2^ = 80%	Critically low confidence
Bermejo (2019)	Butter	High vs. low	*n* = 5 (2 cohorts, 3 CC)	Bladder RR 1.00 (0.95–1.06), *I*^2^ = 6%	Critically low confidence
Sun (2014)	Butter	High vs. low	*n* = 3 (1 cohort, 2 CC)	Gastric RR 1.35 (0.88–2.08), *I*^2^ = 55%	Critically low confidence
Sun (2014)	Margarine	High vs. low	*n* = 3 (2 cohorts, 1 CC)	Gastric RR 1.04 (0.51–2.21), *I*^2^ = 71%	Critically low confidence
***Total mortality***					
Schwingshackl (2014)	Olive oil	Highest vs. lowest	*n* = 5 cohorts	RR 0.77 (0.71–0.84), *I*^2^ = 0%	Critically low confidence
Pimpin (2016)	Butter	per 14 g/day increment	Two publications including *n* = 9 cohorts	RR 1.01 (1.00–1.03), *I*^2^ = 0%	Low confidence
O ’sullivan (2013)	Butter	High vs. low	*n* = 4 cohorts	RR 0.96 (0.85–1.08), *I*^2^ = 78%	Low confidence
***Lipids and blood pressure***					
Duarte (2021)	Butter	Coconut oil, olive oil, soybean oil, palm oil	*n* = 3 interventions	Butter increased LDL-cholesterol in one study (compared with both coconut oil and olive oil) but not in two other studies (compared with olive oil, coconut oil, soybean oil, palm oil)	High confidence
Schwingshackl (2018)	Butter	Safflower oil, sunflower oil, rapeseed oil, hempseed oil, flaxseed oil, corn oil, olive oil, soybean oil, palm oil, coconut oil, beef fat, lard	*n* = 1–7 interventions for each comparison (or indirect effect derived through network meta-analysis)	Butter increase LDL-cholesterol compared to almost all comparisons	High confidence
Hisham (2020)	Palm oil	Diets rich in MUFA or PUFA	*n* = 1 parallel intervention study and *n* = 16 cross-over for MUFA; *n* = 3 parallel intervention studies and *n* = 5 cross-over for PUFA	Compared to MUFA-rich diets, palm oil increased LDL-cholesterol in cross-over studies but decreased LDL-cholesterol in the one parallel study. Compared to PUFA-rich diets, palm oil increased LDL-cholesterol in cross-over studies but not in parallel studies	Low confidence
Voon (2019)	Palm olein	SFA-rich oils (coconut oil, lard), MUFA-rich oils (olive oil, peanut oil, canola oil, high oleic sunflower oil), PUFA-rich oils (soybean oil), all other oils combined	*n* = 5 for SFA-rich oils, *n* = 9 for MUFA-rich oils, *n* = 2 for PUFA-rich oils, *n* = 16 for all other oils combined	Palm olein decrease LDL-cholesterol compared to SFA-rich oils, no difference compared to MUFA- or PUFA-rich oils or all other oils combined	Critically low confidence
Teng (2019)	Coconut oil	Animal fats (butter, beef fat), MUFA-rich oils, PUFA-rich oils, MUFA+PUFA-rich oils, all plant oils combined, all other fats/oils combined	*n* = 17 for all other fats/oils combined, *n* = 4 for animal fats, *n* = 5 for MUFA-rich oils, *n* = 5 for PUFA-rich oils, *n* = 10 for MUFA+PUFA-rich oils, *n* = 13 for all plant oils combined	Coconut oil increase LDL-cholesterol compared to all plant oils combined, PUFA-rich oils, MUFA+PUFA-rich oils but no difference compared to MUFA-rich oils or all other fats/oils combined. Coconut oil decrease LDL-cholesterol compared to animal fats	Low confidence
Neelakantan (2020)	Coconut oil	Palm oil, non-tropical vegetable oils	*n* = 4 for palm oil, *n* = 16 for non-tropical vegetable oils	Coconut oil increase LDL-cholesterol compared to both palm oil and non-tropical vegetable oils	High confidence
Ghobadi (2019)	Olive oil	SFA-rich oils, n-3-rich oils, n-6-rich oils, all other plant oils combined	*n* = 3 for SFA-rich oils, *n* = 12 for n-3-rich oils, *n* = 10 for n-6-rich oils, *n* = 24 for all other plant oils combined	Olive oil increase LDL-cholesterol compared to all other plant oils combined, no difference compared to SFA-rich-, n-3-rich- or n-6-rich oils	Low confidence
Schwingshackl (2019)	Type of olive oil	Refined olive oil, low polyphenol olive oil	*n* = 1–4 comparisons	High polyphenol olive oil decrease LDL-cholesterol compared to low polyphenol olive oil, no difference when low- or high polyphenol olive oil where compared to refined olive oil	High confidence
George (2019)	Olive oil, high polyphenol	Low polyphenol olive oil	*n* = 5 in healthy populations, *n* = 5 in CVD population	High polyphenol olive oil decrease LDL-cholesterol in healthy but not in CVD or total population	Low confidence
Amiri (2020)	Canola oil	Olive oil, sunflower oil, SFA, all other oils combined	*n* = 9 for olive oil, *n* = 11 for sunflower oil, *n* = 10 for SFA, *n* = 35 for all other oils combined	Canola oil decrease LDL-cholesterol in all comparisons	High confidence
Yang (2021)	Flaxseed oil	Sunflower oil, safflower oil, corn oil	*n* = 9 RCTs	Flaxseed oil had no differential impact on LDL-, HDL- or total cholesterol, nor on triglycerides	Critically low confidence
Ursoniu (2016)	Flaxseed oil	Not specified	*n* = 6	Flaxseed oil decrease diastolic- but not systolic blood pressure	Critically low confidence
Huth (2015)	High oleic vegetable oil	SFA, TFA, PUFA	*n* = 23 for SFA, *n* = 6 for TFA, *n* = 11 for PUFA	Compared to SFA, high oleic vegetable oil decreased LDL-cholesterol in 20 of 23 comparisons and in all comparisons with TFA. Inconclusive when compared to PUFA	Critically low confidence
***Inflammation***					
Schwingshackl (2015)	Olive oil	CLA capsules, n-3 FA capsules, low-fat diet, National Cancer Institute diet, flaxseed oil, coconut/palm oil, healthy diet, olive oil without polyphenols, or Mediterranean diet plus nuts	RCTs *n* = 15 CRP, IL-6 *n* = 7, endothelial function *n* = 8	CRP (−0.64 mg/L [−0.96, −0.31], *I*^2^ = 66%), IL-6 (−0.29 [−0.7, −0.02], *I*^2^ = 62%), Endothelial function (0.76% [0.27, 1.124], *I*^2^ = 26%)	Low confidence
Yang (2021)	Flaxseed oil	Sunflower oil, safflower oil, corn oil	RCTs *n* = 3 CRP, *n* = 3 hs-CRP, *n* = 3 IL-6, *n* = 5 TNF-α	CRP (−0.10 mg/L(−0.46, 0.26), *I*^2^ = 0%), hs-CRP (−1.54 mg/L, (−2.59, −0.49), *I*^2^ = 33%), IL-6 (−0.35 pg/mL (−0.67, −0.03), *I*^2^ = 52%), TNF-α no effect	Critically low confidence
Rahimlou (2019)	Flaxseed oil	Olive oil, sunflower, safflower, MCT, placebo	RCTs *n* = 7 CRP, *n* = 10 hs-CRP, *n* = 4 TNF-α, *n* = 3 IL-6	CRP (−0.56 mg/L [−1.44, 0.30], *I*^2^ = 85.7%), hs-CRP (−0.81 mg/L [−1.85, 0.22], *I*^2^ = 91.1%), TNF-α (0.26 pg/mL [−0.04, 0.57], *I*^2^ = 0%), IL-6 (−0.43 pg/mL [−1.17, 0.31], *I*^2^ = 0%)	High confidence
Ursoniu (2019)	Flaxseed oil	Placebo	*n* = 7 RCTs	CRP (−0.67 mg/L [−2.00, 0.65], *I*^2^ = 83%)	Critically low confidence
Ren (2016)	Flaxseed oil	Olive oil, MUFA enriched olive oil, sunflower oil, safflower oil, soy bean oil	RCTs *n* = 8 (6 parallel, 2 crossover)	CRP (0.39 mg/L [−0.09, 0.87], *I*^2^ = 56%)	Low confidence
***Glucose metabolism***					
Dhanasekara (2021)	Coconut oil	Other oils, fats or non-use	*n* = 11 RCTs	Fasting plasma glucose 2.05 mg/dL (−0.14, 4.25), *I*^2^ = 7%; Fasting insulin 0.31 mIU/L (−2.59, 3.20), *I*^2^ = 28%; HOMA-IR 0.55 (0.00–1.10), *I*^2^ = 64%); HOMA-β 17.09 (−44.31, 10.13), *I*^2^ = 82%	High confidence
Zulkiply (2019)	Palm oil	Partially hydrogenosed soy-bean oil, soy-bean oil, olive oil, canola oil, rapeseed oil, sunflower oil	*n* = 8 RCTs (1 parallel, 7 crossover) with 2 studies in each comparison for fasting plasma glucose, *n* = 6 for fasting insulin	No effects on fasting plasma glucose or fasting insulin	Critically low confidence
***Body weight***					
Yang (2021)	Flaxseed oil	Sunflower oil, safflower oil, corn oil	RCTs *n* = 4 for weight, *n* = 8 for BMI, *n* = 2 for waist-to-hip ratio, *n* = 4 for waist circumference	Waist circumference (−1.61 cm, (−2.69, −0.53), *I*^2^ = 50%), no effect on weight, BMI, waist-to-hip ratio	Critically low confidence
Raeisi-Dehkordi (2019)	Canola oil	Other vegetable oils, fish oil or control diet	*n* = 22 RCTs	Weight (−0.3 kg (−0.52, −0.08), *I*^2^ = 0%), BMI (−0.07 kg/m2 (−0.27, 0.12), *I*^2^ = 0%), no effects on other anthropometric indexes	Low confidence
***Age-related macular degeneration***					
Dinu (2019)	Butter	higher vs. lower	*n* = 2 cohorts	RR 1.04 (0.93–1.16)	Low confidence
	Margarine	higher vs. lower	*n* = 3 studies	RR 1.05 (0.91–1.21)	
	Oils	higher vs. lower	*n* = 2 cohorts	RR 1.10 (0.98–1.23)	
***Endometriosis***					
Qi (2021)	Butter	high vs. low	*n* = 3 (1 cohort, 2 CC)	RR 1.27 (1.03–1.55), *I*^2^ = 0%	Critically low confidence
***Main components of metabolic syndrome***					
Pastor (2021)	Olive oil	Other oils	*n* = 17 studies for body composition, *n* = 12 for glycemic profile, *n* = 15 for blood pressure	Body composition −0.02 (−0.10, 0.05), *I*^2^ = 18%; glycemic profile −0.00 (−0.12, 0.11), *I*^2^ = 40%; blood pressure −0.00 (−0.06, 0.05), *I*^2^ = 37%	Critically low confidence
***Parkinson’s disease***					
Jiang (2014)	Butter	highest vs. lowest	*n* = 2 cohorts	RR 0.76 (0.51–1.13), *I*^2^ = 0%	Critically low confidence

**Table 2:** List of excluded studies

PMID	1st author	Year	Title	Exposure	Reason for excluding
30899527	Wanders	2019	Plant-derived polyunsaturated fatty acids and markers of glucose metabolism and insulin resistance: a meta-analysis of randomized controlled feeding trials	Plant-derived PUFA	Fatty acid level
29228348	Mohammadi-Sartang	2017	Flaxseed supplementation on glucose control and insulin sensitivity: a systematic review and meta-analysis of 25 randomized, placebo-controlled trials	Flaxseed supplementation	Primarily patients
33264277	Neuenschwander	2020	Intake of dietary fats and fatty acids and the incidence of type 2 diabetes: A systematic review and dose-response meta-analysis of prospective observational studies	Animal/vegetable fat	Sources of fat not specified
27074618	Gouveia Lde	2016	Effects of the Intake of Sesame Seeds (Sesamum indicum L.) and Derivatives on Oxidative Stress: A Systematic Review	Sesame oil	Patients
31373097	Afroz	2019	A systematic review on antioxidant and antiinflammatory activity of Sesame (Sesamum indicum L.) oil and further confirmation of antiinflammatory activity by chemical profiling and molecular docking	Sesame oil	Not relevant
26595162	Cao	2016	Dietary total fat and fatty acids intake, serum fatty acids and risk of breast cancer: A meta-analysis of prospective cohort studies	Animal/vegetable fat	Sources of fat not specified
20697723	Liu	2011	Is dietary fat associated with the risk of colorectal cancer? A meta-analysis of 13 prospective cohort studies	Animal/vegetable fat	Sources of fat not specified
26568366	Jiang	2015	Dietary fat intake and endometrial cancer risk: dose-response meta-analysis of epidemiological studies	Animal/vegetable fat	Sources of fat not specified
26515595	Hou	2015	Dietary fat and fatty acid intake and epithelial ovarian cancer risk: evidence from epidemiological studies	Animal/vegetable/dairy based fat	Sources of fat not specified
26402223	Han	2015	Dietary Fat Intake and Risk of Gastric Cancer: A Meta-Analysis of Observational Studies	Animal/vegetable fat	Sources of fat not specified
28935150	Farinetti	2017	Mediterranean diet and colorectal cancer: A systematic review	Olive oil	No SR
30484282	Abdelhamid	2018	Polyunsaturated fatty acids for the primary and secondary prevention of cardiovascular disease	PUFA	Fatty acid level
26528631	Grosso	2017	A comprehensive meta-analysis on evidence of Mediterranean diet and cardiovascular disease: Are individual components equal?	Mediterranean diet	Dietary pattern
24472634	Hu	2014	Dairy foods and risk of stroke: a meta-analysis of prospective cohort studies	Butter	More recent SR available
31089743	Cavero-Redondo	2019	Milk and Dairy Product Consumption and Risk of Mortality: An Overview of Systematic Reviews and Meta-Analyses	Butter	Covered by other SR
24717342	Fattore	2014	Palm oil and blood lipid-related markers of cardiovascular disease: a systematic review and meta-analysis of dietary intervention trials	Palm oil	Fatty acid level
25995283	Sun	2015	Palm Oil Consumption Increases LDL Cholesterol Compared with Vegetable Oils Low in Saturated Fat in a Meta-Analysis of Clinical Trials	Palm oil	Covered by more recent SR
26055128	Hohmann	2015	Effects of high phenolic olive oil on cardiovascular risk factors: A systematic review and meta-analysis	Olive oil, high polyphenol	Covered by more recent SR
27882320	Rondanelli	2016	MediterrAsian Diet Products That Could Raise HDL-Cholesterol: A Systematic Review	Olive oil	Covered by more recent SR
30725578	Ma	2016	Virgin Coconut Oil and its Cardiovascular Health Benefits	Coconut oil	Primarily animals
30381009	Ghobadi	2019	Effects of Canola Oil Consumption on Lipid Profile: A Systematic Review and Meta-Analysis of Randomized Controlled Clinical Trials	Canola oil	Covered by more recent SR
33762150	Schoeneck	2021	The effects of foods on LDL cholesterol levels: A systematic review of the accumulated evidence from systematic reviews and meta-analyses of randomized controlled trials	Many foods	Umbrella review
35043479	Sohouli	2021	Consumption of sesame seeds and sesame products has favorable effects on blood glucose levels but not on insulin resistance: A systematic review and meta-analysis of controlled clinical trials	Sesame products	Patients
34527059	Musazadeh	2021	Flaxseed Oil Supplementation Augments Antioxidant Capacity and Alleviates Oxidative Stress: A Systematic Review and Meta-Analysis of Randomized Controlled Trials	Flaxseed oil	Patients
31464396	Wang	2019	Effect of palm oil consumption on plasma lipid concentrations related to cardiovascular disease: a systematic review and meta-analysis	Palm oil	Covered by more recent SR
27713961	Fabiani	2016	Anti-cancer properties of olive oil secoiridoid phenols: a systematic review of in vivo studies	Olive oil phenols	Short / not specified duration of intervention

## Diet intake in Nordic and Baltic countries

It is challenging to directly compare the intake of fats and oils between the Nordic and Baltic countries as food groupings and assessment methods are not harmonized. Food group definitions, and thus level of information, differ between countries. Furthermore, data was collected at different time points (from 2010 to 2020) using different instruments (2 × 24 h recall, 4-day web-based food record, and 7-day food record), and participation rate ranged between 33 and 90%. Finally, contribution of alcohol to total energy intake was not included in Latvia and Lithuania. However, based on available data (9) it appears that the intake of fats and oils is higher in Denmark (men: 47 g/day, women: 35 g/day) and Finland (men: 53 g/day, women: 38 g/day) than in Norway (men: 39 g/day, women: 24 g/day) and Iceland (men: ~20 g/day, women: ~15 g/day). Data for Sweden only includes ‘spreads on bread’ (being 13 g/day for men, 10 g/day for women) and is thus difficult to compare to the other countries. In the Baltic countries, intake appears higher in Estonia (men: ~26 g/day, women: 19 g/day) than in Latvia (men: 13 g/day, women: 11 g/day) and Lithuania (men:12 g/day, women: 9 g/day). However, there is a large standard deviation/range in all countries.

## Health outcomes relevant for Nordic and Baltic countries

### Cardiovascular disease

#### Olive oil

In a meta-analysis from 2014 (10) based on seven cohort studies (population ranged from *n* ~3,300 to *n* ~41,000 with follow-up durations ranging from 3.7 to 11.3 years), the intake of olive oil (top vs. bottom third) was associated with a 28% reduction in the risk for combined cardiovascular events (relative risk [RR] 0.72 [95% confidence interval [CI]: 0.57–0.91]), although with considerable heterogeneity (*I*^2^ = 75%). Results in the same direction were observed at the nutrient level for both ‘all MUFA combined’ (RR 0.91 [0.86–0.96], 30 studies) and ‘MUFA:SFA ratio’ (RR 0.93 [0.86–1.01], six studies). For CVD mortality, a risk reduction of 30% was indicated for higher compared to lower intake; however, substantial heterogeneity (*I*^2^ = 71%) caused by one study resulted in a statistically non-significant result (RR 0.70 [0.48–1.03]). Similar results, although of smaller magnitudes, were observed at the nutrient level for both ‘all MUFA combined’ (RR 0.88 [0.80–0.96], *n* = 14 studies) and ‘MUFA:SFA ratio’ (RR 0.91 [0.83–0.99], four studies). When looking at the risk of stroke separately, the intake of olive oil (highest vs. lowest third) was associated with a 40% reduction in risk (RR 0.60 [0.47–0.77]), without heterogeneity (*I*^2^ = 0%) but based on only two cohorts. When looking at the risk of coronary heart disease (CHD) separately, the intake of olive oil (highest vs. lowest third) was not associated with risk (RR 0.80 [0.57–1.14]), based on four cohorts with considerable heterogeneity (*I*^2^ = 77%). Another meta-analysis from 2014 based on the same data observed similar results for both stroke and CHD when exposure was expressed as ‘per 25 g/day increment’.

The beneficial effect of olive oil on CVD indicated by meta-analyses of observational studies is supported by the ~5 year-long PREDIMED trial, where the risk of CVD (composite of myocardial infarction, stroke and death from cardiovascular causes) decreased by ~30% in the group receiving extra-virgin olive oil in the context of a Mediterranean diet compared to the control group (receiving advice on a low-fat diet).

#### Butter

In a meta-analysis from 2016 (11) based on cohort studies from Sweden, USA, Finland, and the Netherlands, there was no association between the intake of butter (per 14 g/day increment) and the risk for total CVD (RR 0.99 [0.96–1.02], *n* = 2 cohorts with *n* = 6,051 cases), stroke (RR 1.01 [0.98–1.03], *n* = 3 cohorts with *n* = 5,229 cases), CHD (RR 0.99 [0.96–1.03], *n* = 3 cohorts with *n* = 4,484 cases) or any CVD outcome (RR 1.00 [0.98–1.02], *n* = 4 cohorts with *n* = 9,783 cases), without heterogeneity (*I*^2^ = 0% for all). For stroke specifically, another meta-analysis from 2016 (12) based on largely the same data also did not observe any association (RR 1.00 [0.99–1.01] per 10 g/day increment). The null association between butter and CVD reported above is supported by findings by the US Dietary Guidelines Advisory Committee 2020 (8).

#### Palm oil

In a SR from 2018 (13), one single case-control study (*n* = 2,111 cases) from Costa Rica was identified comparing the intake of palm oil with soybean oil and ‘other oils’ regarding the risk of myocardial infarction. The results indicated that palm oil was associated with increased risk compared with ‘other oils’ (RR 1.26 [1.02–1.55]) and soybean oil with low (5%) levels of trans-fat (RR 1.33 [1.09–1.62]) but not when compared with soybean oil with high (22%) levels of trans-fat (RR 1.16 [0.86–1.56]). The certainty of evidence was graded as ‘very low’.

### Type 2 diabetes

#### Olive oil

In a meta-analysis from 2017 (14) based on four cohort studies including 19,081 type 2 diabetes (T2D) cases among 183,370 participants from the US and Europe, high olive oil consumption compared to low showed a 16% reduced risk of T2D (RR 0.84 [0.77–0.92], *I*^2^ = 22%) when the duration of follow-up ranged from 4 to 22 years. Further, in a dose-response meta-analysis, 10 g increase of olive oil daily intake was associated with 9% lower risk (RR 0.91 [0.87–0.95]). In addition, a non-linear association was observed so that olive oil intake showed stronger protective association up to the 13 g daily intake (14). However, the quality of meta-evidence of these studies was graded as low (14). Moreover, a SR from 2015 (15) reported that olive oil was found to be protective from T2D in two randomized controlled trials (RCTs) among Spanish adults that compared Mediterranean diet supplemented with virgin olive oil or nuts to a control group who had been advised to comply with a low-fat diet (PREDIMED trial) (16, 17). In the olive oil group (*n* = 139; median age of participants = 67 years), the risk of T2D was 51% lower than in low-fat diet group (*n* = 134) during the median follow-up of 4 years (hazard ratio [HR] 0.49 [0.25–0.97]) (16). Also, later data based on a larger number of participants (*n* = 3,541) aged 55 to 80 years and having high cardiovascular risk showed protective effect for olive oil (HR 0.60 [0.43–0.85]) (17).

#### Butter

A meta-analysis from 2016 (11) based on 11 country-specific cohorts consisting of European and US populations, including altogether 201,628 participants with 23,958 cases, showed that 14 g increase of daily butter consumption was associated with lower risk of type 2 diabetes (RR 0.96 [0.93–0.99], *I*^2^ = 47%).

### Cancer

#### Olive oil and vegetable oils

A meta-analysis from 2022 based on 45 large studies, including eight cohort studies with 12,461 cases in a total cohort of 929,771 subjects and 37 case-control studies with 17,369 cases and 28,294 controls, summarized that high olive oil consumption compared to low was associated with 31% reduced risk of having any type of cancer (RR 0.69 [0.62–0.77], *I*^2^ = 75%). However, when cohort and case-control studies were analyzed separately, there was no association among eight cohort studies (RR 0.90 [0.77–1.05], *I*^2^ = 52%), although a protective association was observed among 37 case-control studies (OR 0.65 [0.57–0.74], *I*^2^ = 67%). A protective overall association between higher olive oil intake and any type of cancer was seen both among Mediterranean (RR 0.69 [0.60–0.79], *I*^2^ = 70%) and non-Mediterranean participants (RR 0.49 [0.34–0.71], *I*^2^ = 49%) (18).

Similarly, high olive oil consumption was related to lower risk for developing a gastrointestinal cancer, that is, colorectal, esophageal, gastric, and pancreatic cancer in overall meta-analysis that combined two cohort and 13 case-control studies (RR 0.77 [0.66–0.89], *I*^2^ = 41%) as well as among 13 case-control studies (RR 0.72 [0.61–0.85], *I*^2^ = 39%), whereas a protective association was not observed in a meta-analysis of cohort studies (RR 0.97 [0.75–1.24], *I*^2^ = 21%) (18). Further, high intake of olive oil was protectively associated with upper aero-digestive cancer (laryngeal, nasopharyngeal, and oral/pharyngeal) among six case-control studies (RR 0.74 [0.60–0.91], *I*^2^ = 33%) and urinary tract cancer (prostate and bladder) in a meta-analysis including six case-control studies (RR 0.46 [0.29–0.72], *I*^2^ = 73%).

Furthermore, in a meta-analysis from 2022 (18) based on three cohort and 11 case-control studies, high versus low consumption of olive oil showed 33% lower risk of breast cancer (RR 0.67 [0.52–0.86], *I*^2^ = 83%). However, in a subgroup analysis by study design, this protective association was observed only among 11 case-control studies (RR 0.63 [0.45–0.87], *I*^2^ = 80%), while there was no association within the three cohort studies (RR 0.67 [0.29–1.56], *I*^2^ = 78%). In another meta-analysis from 2015 (19) based on five cohorts and 11 case-control studies (11,161 cases in over 150,000 women from Europe, the US and Iran), the highest versus lowest category of intake of vegetable oils, including olive, safflower seed, peanut, soya, corn, or mixed oil, was not associated with the risk of breast cancer (OR 0.88 [0.77–1.01]). However, there was substantial heterogeneity among studies (*I*^2^ = 74%). Neither dose-response meta-analysis (*n* = 12 studies with 6,253 cases in 73,842 women) did show any association between 10 g increment of daily vegetable oil intake and the risk of breast cancer (OR 0.98 [0.95–1.01]) (19). Subgroup analysis showed no indication that menopause or hormone receptor status may modify the association between vegetable oils and the risk of breast cancer (19).

#### Butter and margarine

In a meta-analysis of two large cohort studies including participants from Europe (1,303 cases in 301,107 women) and the US (1,531 cases in 205,863 women), consumption of butter was associated with increased risk of endometrial cancer (highest vs. lowest category RR 1.14 [1.03–1.26], *I*^2^ = 3%) (20). Also, high intake of butter showed association with increased risk of non-Hodgkin lymphoma in a meta-analysis of four case-control studies (RR 1.31 [1.04–1.65], *I*^2^ = 37%) (21). In contrast, butter intake showed no associations with the risk of esophageal squamous cell carcinoma (*n* = 4 case-control studies with severe heterogeneity *I*^2^ = 80%; high vs. low 1.77 [0.84–3.75]) (22), bladder cancer (*n* = 2 cohorts, 3 case-control studies; RR 1.00 [0.95–1.06], *I*^2^ = 6%) (23), or gastric cancer (*n* = 1 cohort, 2 case-control studies; highest vs. lowest category RR 1.35 [0.88–2.08], *I*^2^ = 55%) (24). Margarine was not associated with gastric cancer (*n* = 2 cohorts, 1 case control; highest versus lowest category RR 1.04 [0.51–2.21], *I*^2^ = 71%) (24).

### Other health outcomes

A meta-analysis from 2014 (25) investigated the association between dairy foods and risk of Parkinson’s disease. Based on three effect sizes from two prospective cohorts (one from Finland, one from USA), the intake of butter (highest vs. lowest category) was not associated with the risk of Parkinson’s disease (RR 0.76 [0.51–1.13], *I*^2^ = 0.0%).

A meta-analysis from 2019 (26) investigated the association between food groups and risk of age-related macular degeneration in prospective cohort studies. The risk of developing age-related macular degeneration was not associated with the intake (higher vs. lower) of butter (RR 1.04 [0.93–1.16], *n* = 2 studies), margarine (RR 1.05 [0.91–1.21], *n* = 3 studies) or oils (RR 1.10 [0.98–1.23], *n* = 2 studies).

In a meta-analysis from 2021 (27) based on one prospective cohort and two case-control studies, higher butter consumption was associated with a 27% increased risk of endometriosis in the females compared to lower intake (1.27 [1.03–1.55], *I*^2^ = 0%). Studies included 1,173 endometriosis cases from the US and Europe.

A meta-analysis from 2021 (28) investigated the effects of olive oil on the main components of metabolic syndrome (obesity, insulin resistance or glucose intolerance, dyslipidemia, and high blood pressure) in healthy adults or subjects with at least one component related to metabolic syndrome. Olive oil had no effect on body composition (−0.02 [−0.10, 0.05], *I*^2^ = 18%) or glycemic profile (0.04 [−0.10, 0.18], *I*^2^ = 40%) or blood pressure (−0.00 [−0.06, 0.05], *I*^2^ = 37%) compared to other oils (e.g. sunflower oil, palm olein, cocoa butter, fish oil, cottonseed oil, butter, coconut oil, corn oil, soybean oil, safflower oil, canola oil).

### Total mortality

#### Olive oil

In a meta-analysis from 2014 (10) based on five cohort studies (population ranged from n ~3,300 to n ~41,000 with follow-up durations ranging from 3.7 to 11.3 years), the intake of olive oil (top vs. bottom third) was associated with a 23% reduction in risk for all-cause mortality (RR 0.77 [0.71–0.84]). No heterogeneity was observed (*I*^2^ = 0%). Similar results, although of smaller magnitudes, were observed at the nutrient level for both ‘all MUFA combined’ (RR 0.89 [0.83–0.96], *n* = 17 studies) and ‘MUFA:SFA ratio’ (RR 0.90 [0.82–1.00], *n* = 10 studies).

In the PREDIMED trial, supplementation with extra-virgin olive oil in the context of a Mediterranean diet was associated with a non-significant 10% reduction in risk for total mortality (HR 0.90 [0.69–1.18]).

#### Butter

In a meta-analysis from 2016 based on two large studies consisting of European populations (nine country-specific cohorts, ~10 years of follow-up, ~28,000 events), the intake of butter was associated with a 1% increased risk for all-cause mortality per 14 g/day increment (RR 1.01 [1.00–1.03]) without heterogeneity (*I*^2^ = 0%). A previous meta-analysis from 2013 (29) based on partly the same data (one overlapping study) observed no association between intake of butter and all-cause mortality (RR 0.96 [0.85–1.08] per each additional serving/week), with considerable heterogeneity (*I*^2^ = 78%).

## Mechanisms

### Blood lipids and blood pressure

#### Butter

Butter increases LDL-cholesterol when compared to basically all other fats/oil, as demonstrated in a SR from 2021 (30) and a network meta-analysis from 2018 (31). The difference in LDL-cholesterol (−0.25 to −0.42 mmol/L per 10 E% isocaloric exchange) is largest when butter is compared to oils rich in unsaturated fats (safflower oil, sunflower oil, rapeseed oil, flaxseed oil, corn oil, olive oil, and soybean oil), but butter increases LDL-cholesterol also in comparison to other sources of SFA (coconut oil, palm oil, and beef fat). Similar effects were observed for total cholesterol. Fewer differences are observed for triglycerides and high-density lipoprotein [HDL]-cholesterol. However, sunflower oil, soybean oil, and palm oil were observed to decrease triglycerides compared to butter (−0.04 to −0.06 mmol/L per 10 E% isocaloric exchange), and coconut oil was observed to increase HDL-cholesterol compared to butter (0.04 mmol/L per 10 E% isocaloric exchange).

#### Palm oil

The most recent meta-analysis (32) included studies using palm oil or palm olein, but not studies using palm stearin, palm kernel oil or red palm oil. Compared to MUFA-rich diets, palm oil increased LDL-cholesterol (0.24 mmol/L [0.06–0.42]) when based on *n* = 16 cross-over studies including a total of *n* = 365 participants, but decreased LDL-cholesterol (−0.28 mmol/L [−0.53 to −0.03]) when based on one parallel study including 60 participants. Compared to PUFA-rich diets, palm oil increased LDL-cholesterol (0.26 mmol/L [0.06–0.45]) when based on five cross-over studies including a total of 114 participants but not when based on three parallel studies including a total of 152 participants (0.54 mmol/L [−0.45 to 1.52]). Similar results, overall, were observed for total cholesterol. Palm oil increased HDL-cholesterol compared to PUFA-rich diets, both when based on five cross-over studies (0.08 mmol/L [0.01–0.15]) and when based on three parallel studies (0.08 mmol/L [0.00–0.16]). Compared to MUFA-rich diets, there was no difference in HDL-cholesterol when based on 16 cross-over studies (0.03 mmol/L [−0.01 to 0.07]), but palm oil increased HDL-cholesterol when based on one parallel study (0.18 mmol/L [0.09–0.27]). Finally, palm oil had no differential effect on apoA1 or apoB when compared to either MUFA-rich diets (*n* = 3 cross-over studies) or PUFA-rich diets (*n* = 2 cross-over studies and *n* = 1 parallel study).

A previous meta-analysis (3) included only studies using palm olein and found that palm olein decreased LDL-cholesterol when compared to other SFA-rich oils/fats (coconut, lard) (−0.50 mmol/L [−0.70 to −0.30]), but there was no difference when compared to MUFA-rich oils (olive oil, peanut oil, canola oil, high-oleic sunflower oil), PUFA-rich oils (soybean oil) or ‘all other oils’. Results were similar for total cholesterol whereas no differential effect was found for triglycerides. Palm olein increased HDL-cholesterol when compared to MUFA-rich diets (0.04 mmol/L [0.00 to 0.07]) and decreased HDL-cholesterol when compared to other SFA-rich oils (−0.06 mmol/L [−0.11 to −0.00]), whereas no differential effects were found when compared to PUFA-rich oils or ‘all other oils’.

#### Coconut oil

A meta-analysis from 2019 (33) observed that coconut oil decreased LDL-cholesterol when compared to animal fats (butter, beef fat) (−0.37 [−0.69 to −0.05], *n* = 4), but increased LDL-cholesterol when compared to ‘all plant oils’ (0.26 [0.09 to 0.43], *n* = 13). Similarly, coconut oil increased LDL-cholesterol when compared to MUFA+PUFA-rich oils (0.34 [0.13 to 0.54], *n* = 10) as well as when compared to PUFA-rich oils only (0.43 [0.15 to 0.72], *n* = 5), but there was no difference when compared to MUFA-rich oils only (0.23 [−0.06 to 0.53], *n* = 5). Interestingly, when the type of coconut oil was considered, virgin/extra virgin coconut oil did not affect LDL-cholesterol compared to other fats/oils combined (−0.08 [−0.35 to 0.20], *n* = 5), whereas unspecified/all types of coconut oil increase LDL-cholesterol (0.20 [0.02 to 0.38], *n* = 12). Results were, overall, similar for total cholesterol whereas coconut oil raised HDL-cholesterol in all comparisons. Fewer differential effects were observed for triglycerides, but coconut oil increased triglycerides when compared to MUFA+PUFA-rich oils (0.21 [0.01 to 0.41], *n* = 10) as well as PUFA-rich oils only (0.31 [0.03 to 0.58], *n* = 5). Similarly, a meta-analysis from 2020 (34) observed that coconut oil increased both LDL and total cholesterol compared to palm oil (*n* = 4) and non-tropical vegetable oils (*n* = 16).

#### Olive oil

In a meta-analysis from 2019 (35) based on 27 randomized trials, olive oil had no differential effect on LDL-cholesterol compared to oils rich in n-3 PUFA (12 studies), n-6 PUFA (10 studies) or SFA (3 studies). When compared to ‘all other plant oils combined’, olive oil was found to increase LDL-cholesterol (*n* = 24 studies), but stratified analyses showed that this effect was only observed in studies lasting a maximum of 30 days (15 studies) and not in studies with longer duration (10 studies). Olive oil increased total cholesterol when compared to oils rich in n-3 PUFA (11 studies), n-6 PUFA (12 studies) and ‘all other plant oils combined’ (26 studies) but not when compared to SFA (3 studies). Olive oil increased both HDL-cholesterol and triglycerides when compared to oils rich in n-3 PUFA and ‘all other plant oils combined’ but not when compared to oils rich in n-6 PUFA or SFA. Finally, olive oil had no differential effect on apoA1 or apoB when compared to ‘all other plant oils combined’ (10 studies).

Another two meta-analyses from 2019 compared the effects of different types of olive oil (36, 37). Olive oil with a high content of polyphenols decreased both LDL-cholesterol and oxidized LDL-cholesterol compared with olive oil with a low content of polyphenols or ‘refined’ olive oil. Furthermore, olive oil with a high content of polyphenols increased HDL-cholesterol when compared with olive oil with low content of polyphenols but not when compared to ‘refined’ olive oil. No differential effect was observed for diastolic blood pressure, but olive oil polyphenols may decrease systolic blood pressure.

#### Canola oil

A meta-analysis from 2020 (38) observed that canola oil decreased LDL-cholesterol when compared to olive oil (−0.17 [−0.29 to −0.04], *n* = 9), sunflower oil (−0.14 [−0.23 to −0.05], *n* = 11), sources of SFA (−0.49 [−0.70 to −0.28], *n* = 10) as well as all ‘other edible oils’ (−0.23 [−0.33 to −0.14], *n* = 35). Results were similar for total cholesterol. Canola oil had no differential effect on HDL-cholesterol or apoA1 compared to olive oil, sunflower oil, sources of SFA or ‘other edible oils’. Canola oil decreased apoB when compared to sources of SFA (−0.09 [−0.16 to −0.02], *n* = 4) and ‘other edible oils’ (−0.03 [−0.06 to −0.01], *n* = 14), but not when compared to olive oil (0.01 [−0.07 to 0.08], *n* = 2) or sunflower oil (0.01 [−0.04 to 0.07], *n* = 3). Canola oil had no differential effect on either systolic or diastolic blood pressure compared to olive oil, sunflower oil, sources of SFA or ‘other edible oils’.

#### Flaxseed oil

Based on a network meta-analysis (a technique for comparing three or more interventions simultaneously) from 2018 (31), the effect of flaxseed oil on LDL-cholesterol is similar to that of most other fats/oils (safflower oil, sunflower oil, rapeseed oil, hempseed oil, corn oil, olive oil, soybean oil, palm oil, coconut oil, beef fat, and lard), but lowers LDL-cholesterol in comparison to butter (−0.37 mmol/L per 10 E% isocaloric exchange). Effects were similar for total cholesterol. Flaxseed oil had no differential effects on HDL-cholesterol or triglycerides compared to any other fats/oils. However, all comparisons are based on only 1–2 studies. Another meta-analysis from 2016 (39) indicates that flaxseed oil may lower diastolic (−4.1 mmHg [−6.8 to −1.4]), but not systolic (−4.6 mmHg [−11.9 to 2.6]), blood pressure; however, the type of comparison was not specified. Finally, a SR and meta-analysis from 2021, based on nine RCTs, found no differential effect of flaxseed oil on total cholesterol, LDL-cholesterol, HDL-cholesterol or triglycerides compared to other oils (sunflower oil, safflower oil, corn oil) in patients with dyslipidemia-related diseases (40).

#### High-oleic vegetable oil

A SR from 2015 (41) investigated the effects of oils rich in oleic acid (primarily high-oleic sunflower oil, but also safflower, olive, and canola oil) on cardiovascular risk factors when compared to other fats and oils (primarily palm oil, but also butter, cocoa butter, lard, and margarines with high content of trans fat). Based on 23 comparisons from 17 crossover interventions (8 studies in hyper- and nine studies in normocholesterolemic subjects), high-oleic oils reduced LDL-cholesterol, apoB, and total cholesterol when compared to oils/fats rich in saturated fat in the majority of comparisons, whereas no differential effect was observed for triglycerides, HDL-cholesterol or apoA1. When compared to trans-fat-containing oils/fats, high-oleic oils decreased total- and LDL-cholesterol, apoB and triglycerides in all or almost all comparisons, whereas HDL-cholesterol and apoA1 were increased in the majority of comparisons. High-oleic oils had no differential effect on any lipoprotein marker compared to oils rich in PUFA.

### Inflammation markers

The largest available meta-analysis from 2015 (42) (*n* = 15 RCTs), including altogether 541 participants in the olive oil groups and 731 in control groups, showed that olive oil compared to control oil or diet reduced levels of c-reactive protein (CRP) (mean difference 0.64 mg/L [−0.96, −0.31], *I*^2^ = 66%). Also, olive oil decreased concentration of interleukin 6 (IL-6) (mean difference −0.29 pg/mL ([−0.7, −0.02], seven RCTs with a total of 416 subjects in olive oil and 441 in control groups, *I*^2^ = 62%)) as well as improved endothelial function (mean difference 0.76% ([0.27–1.124]; eight RCTs with 335 subjects in olive oil and 516 in control groups, *I*^2^ = 26%)). The study duration varied from four to 208 weeks. However, a considerable heterogeneity among studies indicates that results of this meta-analysis should be interpreted with caution.

In a meta-analysis from 2019 based on seven RCTs, flaxseed oil compared to placebo did not affect plasma CRP levels (−0.67 mg/L [−2.00, 0.65], *I*^2^ = 83%). Participants in these studies were healthy, overweight, obese, prediabetic or patients with chronic diseases (43). Also, in two other meta-analyses, flaxseed oil did not affect circulating inflammatory markers (CRP, IL-6, TNF-α, or high sensitive CRP) compared to other vegetable oils (44, 45). In contrast, in a meta-analysis from 2021 including participants with dyslipidemia related diseases, flaxseed oil reduced IL-6 (−0.35 pg/mL [−0.67, −0.03], *I*^2^ = 52%) and high sensitive CRP (−1.54 mg/L [−2.59, −0.49], *I*^2^ = 33%) compared to other vegetable oils (sunflower, safflower or corn), while there was no effect on CRP or TNF-α (40).

### Glucose metabolism

In a meta-analysis from 2021, coconut oil intake compared to meals without coconut oil increased insulin resistance (HOMA-IR mean difference 0.55 [0.00–1.10], *I*^2^ = 64%), but did not affect fasting plasma glucose (mean difference 2.05 mg/dL, [−0.14, 4.25], *I*^2^ = 7%), insulin (mean difference 0.31 mIU/L [−2.59, 3.20], *I*^2^ = 28%) or HOMA-β (mean difference 17.09 [−44.31, 10.13], *I*^2^ = 82%) (46). This meta-analysis consisted of 11 RCTs, in which the number of participants ranged from 9 to 92 in intervention groups and the duration of the interventions varied from 3 to 28 weeks.

In a SR from 2019 (47) results from eight RCTs that compared the effect of palm oil on glucose metabolism in interventions that lasted from 3 to 7 weeks and the number of participants in intervention groups ranged from 15 to 100 participants were presented. Palm oil compared to other vegetable oils was not seen to affect fasting plasma glucose or insulin levels (47).

### Body weight and other anthropometric measures

In a meta-analysis from 2019 (48) based on 22 RCT studies including 1,078 participants, canola oil reduced body weight 0.3 kg (−0.52, −0.08) compared to many other types of vegetable oils, fish oil or control diet in interventions of varying durations from three to 28 weeks. These studies were done in several countries around the world including Finland and Sweden and there was no heterogeneity between the studies (*I*^2^ = 0%). However, canola oil was not observed to affect BMI or other anthropometric measures.

In a meta-analysis from 2021 in participants with dyslipidemia related diseases, flaxseed oil intake did not affect body weight (*n* = 4 RCT), BMI (*n* = 8 RCT) or waist-to-hip ratio (*n* = 2 RCT), but it reduced waist circumference (*n* = 4 RCT) 1.61 cm [−2.69, −0.53], *I*^2^ = 50%, compared to other vegetable oils (40).

## Food-based dietary guidelines

### Summary of evidence

#### Associations with diseases and mortality

- Consumption of olive oil was associated with lower risk of CVD, T2D, and total mortality in prospective cohort studies.
- Consumption of butter was not associated with risk of CVD but it was related to slightly lower risk of T2D and higher risk of total mortality in prospective cohort studies.
- Consumption of olive oil was associated with reduced risk of some cancer types whereas intake of butter was associated with higher risk of certain cancer types. However, the evidence was scarce and mainly based on case-control studies.

#### Effects on risk markers and body weight

- Canola oil decreased low-density lipoprotein [LDL]-cholesterol compared to olive oil, sunflower oil and sources of SFA and also reduced body weight compared to other oils.
- Olive oil did not affect LDL-cholesterol but decreased some inflammation markers compared to other fats and oils. Olive oil with high content of polyphenols may have more beneficial effects on risk markers compared to olive oil with low content of polyphenols.
- Palm oil increased LDL-cholesterol when compared to oils rich in MUFA or PUFA but did not affect glucose or insulin levels.
- Coconut oil increased LDL-cholesterol when compared to other vegetable oils but decreased LDL-cholesterol when compared to animal fats rich in SFA. Coconut oil increased insulin resistance compared to other oils and fats or non-use.
- Flaxseed oil showed some anti-inflammatory effects and reduced waist circumference compared to other vegetable oils but only among participants with dyslipidemia related diseases.
- Butter increased LDL-cholesterol when compared to other fats and oils.

### Reasoning and considerations relevant for setting the FBDGs

Based on the current evidence, vegetable oils rich in unsaturated fat (e.g. olive oil, canola/rapeseed oil) are to be preferred over tropical plant oils containing a high percentage of SFA such as coconut oil and palm oil as well as SFA-rich animal fats like butter. This is in line with several other fat recommendations in national dietary guidelines (e.g. USA 2020–2025 (49), Canada 2018 (50), Netherlands 2015 (51), and Germany 2015 (52)).

Non-tropical vegetable oils are rich in unsaturated fatty acids, and should be used as their dietary source. Particularly, the essential fatty acids, n−6 linoleic acid (LA) and n−3 alpha-linolenic acid (ALA), must be obtained from the diet since the human body is not capable of synthesizing them. Several vegetable oils are good sources of LA, whereas only a few oils are rich in ALA (Table 3). Good sources of ALA are canola/rapeseed, linseed, hempseed, soybean and walnut oils. For example, the recommended daily amount of both n-6 PUFA and n-3 PUFA can be obtained by 2–3 tablespoon of canola/rapeseed oil. In a Nordic context, rapeseed oil may thus be considered as the primary source of added fat due to its nutritional profile as well as being locally produced. However, olive oil may entail additional health benefits beyond fatty acid composition (e.g. polyphenols) (36, 37) and may also be used frequently. Unfortunately, very few studies are available that specifically investigates health effects of rapeseed oil. However, based on individual RCTs, rapeseed oil decreases liver fat content during isocaloric conditions compared to olive oil (53), and a diet rich in canola oil was found to decrease abdominal fat even when compared to diets with higher content of PUFA (blends of corn oil, safflower oil and flaxseed oil) (54). Furthermore, meals rich in rapeseed oil increased 24-h fat oxidation compared to meals rich in palm oil (55), and diets rich in rapeseed oil markedly improve blood lipid profile compared to butter (56, 57). Rapeseed oil has a higher content of phytosterols compared to many other oils (1), perhaps explaining the LDL-cholesterol reducing effect of rapeseed oil compared to both olive oil and sunflower oil demonstrated in a recent meta-analysis (38). Finally, a rapeseed oil-based margarine was used in the Lyon Diet Heart Study, a secondary prevention trial testing an ALA-enriched Mediterranean diet in patients having survived a first acute myocardial infarction (58, 59). A striking protective effect was observed on the risk of recurrence (50–70% reduction) after 4 years of follow-up. Although these positive results cannot be ascribed specifically to rapeseed oil, it further supports the role of rapeseed oil as part of a cardioprotective diet. Vegetable oils are 100% fat and therefore include a high energy content. Although adding healthy plant oils to the diet will improve the overall fat composition of the diet, it will also increase the energy content and density of the diet. Thus, from the perspective of weight management, it is advisable to use healthy plant oils in moderate amounts.

**Table 3:** Fats and oils as dietary sources of essential fatty acids^1^

Fat/oil	Portion	Linoleic acid (n-6 PUFA) g/portion	Alpha-linolenic acid (n-3 PUFA) g/portion
Canola/rapeseed oil	1 Tbsp. (14 g)	3.1	1.5
Olive oil	1 Tbsp. (14 g)	1.5	0.1
Sunflower oil	1 Tbsp. (14 g)	8.7	0.1
Soybean oil	1 Tbsp. (14 g)	7.3	1.0
Linseed oil	1 Tbsp. (14 g)	1.8	7.5
Hempseed oil	1 Tbsp. (14 g)	7.4	2.6
Sesame oil	1 Tbsp. (14 g)	5.8	0.1
Walnut oil	1 Tbsp. (14 g)	7.4	1.5
Palm oil	1 Tbsp. (14 g)	1.3	0.0
Coconut oil	1 Tbsp. (14 g)	0.3	0.0
Butter	1 Tbsp. (15 g)	0.2	0.1
Margarine, 40%	1 Tbsp. (15 g)	1.0	0.5
Margarine, 60%	1 Tbsp. (15 g)	1.9	0.6
Margarine, 70%	1 Tbsp. (15 g)	2.4	0.9
Butter mix, 40%	1 Tbsp. (15 g)	0.6	0.3
Butter mix, 60%	1 Tbsp. (15 g)	0.9	0.3
Butter mix, 75%	1 Tbsp. (15 g)	0.8	0.4
**Examples of recommended quantities**			
Daily minimum intake* with an average expenditure of 2,000 kcal		5.6 g (2.5 E%)	1.1 g (0.5 E%)
Intake with 2,000 kcal during pregnancy and lactation		8.9 g (4.0 E%)	2.2 g (1 E%)

1 For margarine and butter mixes, an average of LA and ALA is calculated. Values are from Finnish, Swedish, and US national food databases.

* Because minimum requirements of cis-PUFA for adults are not known, the estimates are based on threshold intake data from children. The recommendation for essential fatty acids, that is, LA and ALA, is 3E% of which at least 0.5E% should be ALA. Recommended energy percentages were calculated as kcal of 2,000 kcal. Then, recommended intake of LA and ALA as grams were calculated based on that 1 g fat yields 9 kcal energy. For example, calculation for LA: 2.5% of 2,000 kcal = 50 kcal; thus, 50 kcal / 9 kcal = 5.6 g.

Processing of oils, such as extraction method, cold-pressing, heating and refining, has no effect on fatty acid composition of oils but they impact on how other bioactive compounds are preserved (2). Refining, however, removes taste, unwanted compounds, and reduces oxidation products that will induce further oxidation if not removed. Further, cooking temperature, light exposure during storage as well as storage temperature and time influence bioactive compounds in oils since they are sensitive to heating and light. Light and heat are also factors that increase oxidation. In general, oils are suitable for frying and cooking (despite their relatively high content of unsaturated fatty acids) (60), but they should not give smoke at high temperatures when cooked. The temperature during normal pan frying is typically 140–175°C, and negative effects of frying vegetable oils high in unsaturated fatty acids are marginal even at 200 °C for extended time periods (60).

### Data gaps for future research

Further systematic investigations would be desirable of different consumption levels of vegetable oils in relation to disease outcomes, mortality, blood lipids as well as overweight and obesity among both adults and children. Current evidence indicates that the type/version (e.g. high or low in polyphenols) of oil may affect risk markers which suggests importance of degree of processing and advantageous health effects of bioactive components. Further studies are needed to clarify the effect of these minor components on health. Margarines and butter mixes are commonly used products in the Nordic countries; however, there is scarce evidence about their overall health effects compared to other sources of dietary fat. Margarines may differ a lot regarding both fat content and fatty acid composition (i.e. which oils/fats that have been used), and more specific studies are needed. As the oils/fats that are used in margarine production are typically highly refined, the levels of bioactive compounds (e.g. polyphenols) may potentially be lower in margarines compared to less refined versions of the parent oils/fats. On the other hand, many beneficial components could be added in margarines, for example, antioxidants, vitamin D or plant sterols and stanols. Furthermore, oils/fats used in margarine production are often interesterified (i.e. fatty acids are rearranged within the triglycerides) to achieve desirable physical structure/functionality. Although no negative effects of interesterification have been indicated in short-term human studies, the evidence is scarce and more studies are warranted. For similar reasons (physical structure), margarines typically contain a variable fraction of tropical oils (e.g. palm, coconut) influencing both the fatty acid and sustainability profile. However, vegetable oil-based margarines usually contain less saturated and trans-fat (levels of trans-fat are very low to absent) than butter or butter mixes, and could therefore be considered a healthier choice as the fatty acid composition is likely the primary factor determining the overall health effect. Margarines with a higher fat content (compared to lower) are generally better sources of essential fatty acids (Table 3).

### Limitations

The main limitation is that there were no recent qualified SRs available reporting at a food level perspective, and neither was a *de novo* NNR2023 SR performed for this topic. Overall, there were few SRs available, and many included SRs had low or critically low confidence, thus the summarized evidence should be interpreted with caution. Furthermore, only few fats/oils (olive oil, butter) have been investigated in relation to disease outcomes in SRs; for example, there was no SR concerning canola/rapeseed oil and disease outcomes. Thus, evidence of olive oil was emphasized in relation to disease outcomes. Furthermore, there were hardly any SRs that included margarines, butter mixes or shortenings. All studies that filled inclusion criteria were done in adults, and therefore evidence of health effects of fat and oils among vulnerable groups, that is, children, adolescents, pregnant and lactating women could not be taken into consideration. There were no SRs available for several diseases, for example, autoimmune diseases, asthma, and allergies. Most of the evidence on disease outcomes are based on observational data, and although statistical adjustment for potential confounders are typically performed, residual confounding likely remains. Data on dietary intake are most often self-reported, and various degrees of misreporting, and thus misclassification, can be expected. Another general limitation that can be discussed is the statistical modelling of the dietary data, where specific substitution models are generally not available, which may hamper the overall interpretation and advice on practical implementation. Evidence was identified in scientific literature only since 2011 and the search was done in one database. Therefore, there might be some relevant older studies which did not meet inclusion criteria. Furthermore, as the search only included SRs and meta-analyses, there may be individual studies of high quality (not included in any SR) that has not been considered.
